# Metabolic Signature of *FLT3*-Mutated AML: Clinical and Therapeutic Implications

**DOI:** 10.3390/jpm15090431

**Published:** 2025-09-08

**Authors:** Cristina Banella, Gianfranco Catalano, Maura Calvani, Eleonora Candi, Nelida Ines Noguera, Serena Travaglini

**Affiliations:** 1 Department of Pediatric Hematology-Oncology, Meyer Children’s Hospital IRCCS, 50139 Florence, Italy; cristina.banella@meyer.it (C.B.); maura.calvani@meyer.it (M.C.); 2 Department of Biomedicine and Prevention, Tor Vergata University, 00133 Rome, Italy; gfcatal@uniroma2.it; 3 Santa Lucia Foundation, I.R.C.C.S., Neuro-Oncohematology, 00143 Rome, Italy; 4 Department of Experimental Medicine, University of Rome Tor Vergata, 00133 Rome, Italy; candi@uniroma2.it

**Keywords:** Acute Myeloid Leukemia, *FLT3*-ITD, metabolic plasticity, metabolic targeting

## Abstract

Acute Myeloid Leukemia (AML) is a genetically and clinically heterogeneous malignancy marked by poor prognosis and limited therapeutic options, especially in older patients. While conventional treatments such as the “7 + 3” chemotherapy regimen and allogeneic stem cell transplantation remain standard care options, the advent of next-generation sequencing (NGS) has transformed our understanding of AML’s molecular complexity. Among the emerging hallmarks of AML, metabolic reprogramming has gained increasing attention for its role in supporting leukemic cell proliferation, survival, and therapy resistance. Distinct AML subtypes—shaped by specific genetic alterations, including *FLT3*, *NPM1*, and *IDH* mutations—exhibit unique metabolic phenotypes that reflect their underlying molecular landscapes. Notably, *FLT3*-ITD mutations are associated with enhanced reactive oxygen species (ROS) production and altered energy metabolism, contributing to disease aggressiveness and poor clinical outcomes. This review highlights the interplay between metabolic plasticity and genetic heterogeneity in AML, with a particular focus on FLT3-driven metabolic rewiring. We discuss recent insights into how these metabolic dependencies may be exploited therapeutically, offering a rationale for the development of metabolism-targeted strategies in the treatment of *FLT3*-mutated AML.

## 1. Introduction

Acute Myeloid Leukemia (AML) is a highly heterogeneous and aggressive hematologic malignancy that primarily affects older adults [1,2]. Despite recent advances in treatment, AML remains a challenging disease with a poor prognosis, particularly in older adults, with a five-year survival rate below 30% [3]. For many years, the “7 + 3” chemotherapy regimen, consisting of cytarabine and anthracycline, followed by allogeneic stem cell transplantation, has been the standard of care for high-risk AML patients, irrespective of their clinical and molecular characteristics [4]. Recent advancements in next-generation sequencing (NGS) technologies have significantly enhanced our understanding of the molecular landscape of AML [5]. These breakthroughs have facilitated the identification of novel genetic aberrations and altered signaling pathways which hold promise as potential prognostic biomarkers and therapeutic targets. In light of this, significant progress has been made toward achieving higher complete remission (CR) rates, longer relapse-free survival (RFS), and better overall survival (OS) in AML [6].

Among emerging biomarkers, metabolic reprogramming represents one of the major hallmarks of cancer, playing a crucial role in cancer cell growth, migration, and metastatic progression [7]. Since Otto Warburg’s publication in 1920s proposing that cancer cells preferentially use glucose over mitochondrial respiration to generate ATP regardless of oxygen conditions [8,9], numerous studies have revealed the dynamic nature of cancer cell metabolism [10]. These investigations have identified a wide spectrum of metabolic alterations, each associated with distinct cancer types and disease stages. This metabolic diversity underscores the complexity of cancer reprograming, highlighting how these shifts might contribute not only to tumor growth but also to the heterogeneity observed among different tumors and their therapeutic responses [11,12,13].

Metabolic plasticity also plays a pivotal role in leukemia initiation and progression [14]. In AML, genetic and molecular heterogeneity is reflected in the preferential metabolic pathways used by different AML subtypes, shaped by their distinct molecular backgrounds. Indeed, numerous studies have shown that specific mutations and molecular signatures can activate oncogenic signaling pathways, directly contributing to these metabolic adaptations, which are crucial for meeting the energy demands and supporting the proliferation and survival of leukemic cells. Notably, an increased production of reactive oxygen species (ROS) has been linked to the aggressive behavior and poor prognosis of *FLT3*-ITD-mutated AML [15]. Beyond *FLT3* mutations, other recurrent genetic alterations, such as *NPM1*, *IDH1*, and *IDH2* and balanced translocation [16,17,18], including t(8;21)(q22;q22), t(15;17)(q24;q21), and t(16;16)(p13;q24) [19,20,21,22,23], profoundly influence the metabolic profile of leukemic cells. Notably, *IDH* mutations provide evidence of the strong connection between leukemogenesis and metabolism, playing a crucial role in the reprogramming of epigenetic, transcriptional, and biochemical profiles of AML cells [18,24]. This metabolic heterogeneity poses a significant challenge in the context of AML therapy, highlighting the necessity of gaining deeper insight into cancer metabolism for the development of treatment strategies directed toward specific metabolic pathways.

Herein, we provide an in-depth exploration of the role of cellular metabolism in AML, with a particular focus on recent discoveries regarding metabolic alterations associated with *FLT3* mutations. We extensively discuss how these mutations impact cellular energy metabolism, highlighting their influence on leukemic cell survival and proliferation. By examining the latest advances in this field, we aim to elucidate the therapeutic implications of targeting distinct metabolic vulnerabilities associated with *FLT3*-mutated AML. A comprehensive understanding of the metabolic preferences driven by *FLT3* mutations may uncover critical dependencies, paving the way for the development of more effective and subtype-specific treatment strategies [25,26,27,28,29,30].

## 2. Clinical and Molecular Features of *FLT3*-Mutated AML

AML is a complex and rapidly progressing malignancy, where the prognosis and treatment outcome are significantly influenced by the underlying genetic and molecular features. Recent advances in molecular biology, with the widespread use of NGS technologies, have uncovered crucial genetic alterations, allowing for an integrated approach that takes into account clinical and laboratory characteristics. This integrated approach has led to improved prognostic stratification and treatment personalization.

Among the most frequently mutated genes in AML, somatic alterations of the *FLT3* gene are recognized as a distinct nosological entity, identified in approximately 25–30% of de novo AML cases. Accordingly, the European LeukemiaNET (ELN) guidelines recommend *FLT3* testing at the time of initial AML diagnosis to guide treatment decisions and assess patient eligibility for allogeneic hematopoietic stem cell transplantation (allo-HSCT). According to the latest update of the ELN 2022 recommendations, two different types of *FLT3* mutations have been considered clinically relevant: internal tandem duplications (ITD) in the juxtamembrane domain (JM) of the gene and point mutations within the tyrosine kinase domain (TKD) [31,32]. In addition, rare deletions and point mutations affecting the JM domain have also been described, but the biological and prognostic significance of these alterations is still unclear [33,34].

*FLT3* abnormalities, particularly the ITD variant, are associated with a high incidence of relapse and aggressive disease progression, making it a critical target for therapeutic intervention in AML management [35,36,37].

Amidst the ongoing advances in the molecular characterization and prognostic stratification of AML patients, the metabolic profile of leukemic cells remains largely overlooked. While the emerging role of metabolism in tumor initiation, progression, and response to therapy has been increasingly recognized, the precise contribution of these cellular metabolic networks to key cancer cell functions is far from being fully understood.

## 3. Metabolic Dependencies in *FLT3*-Mutated AML

**Figure 1:**
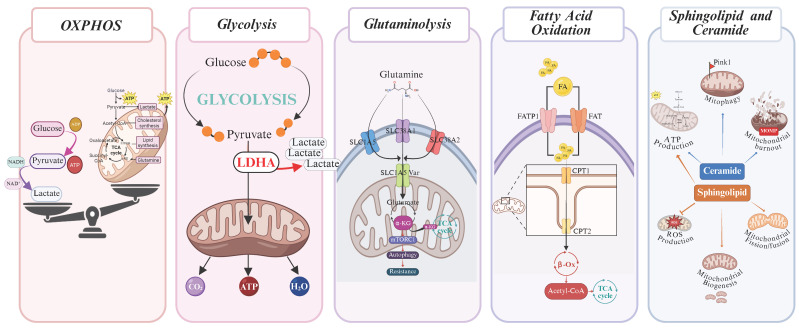
Metabolic dependencies in *FLT3*-ITD-mutated AML. Overview of key altered pathways supporting leukemic cell survival and proliferation.

Hematopoietic stem cells (HSCs), referred to as ‘dormant’ (dHSCs), exhibit low metabolic demands within the bone marrow niche. In their dormant state, HSCs primarily rely on aerobic glycolysis (AG) to maintain low levels of ROS and energy production. However, upon activation, they shift toward oxidative phosphorylation (OXPHOS) and fatty acid oxidation (FAO) to meet increased energy demands [38,39,40]. Similarly, AML cells, particularly those harboring *FLT3* mutations, modulate their metabolic pathways dynamically in response to nutrient availability, microenvironmental stress, and therapeutic pressure (Figure 1) [41,42]. This metabolic flexibility allows cancer cells to sustain proliferation and survival under fluctuating conditions, such as hypoxia or drug exposure, by modulating glycolysis, mitochondrial respiration, and lipid metabolism accordingly [43,44,45,46,47,48]. Therefore, the metabolic features described should be considered adaptable responses rather than fixed characteristics, playing a key role in disease progression and resistance to treatment.

### 3.1. Oxidative Phosphorylation (OXPHOS)

**Figure 2:**
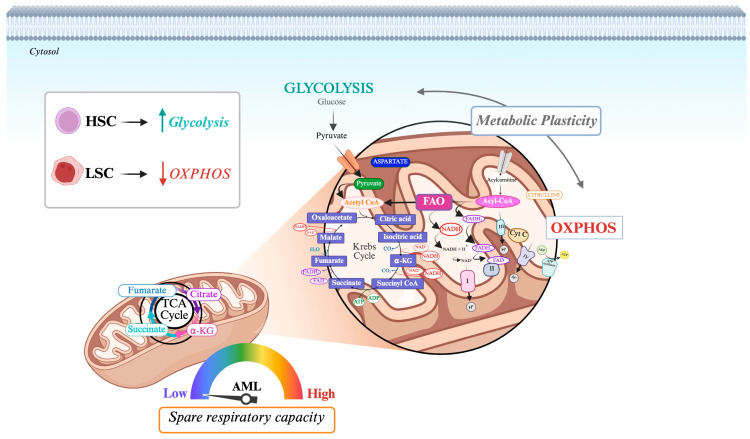
Mitochondrial bioenergetics in *FLT3*-ITD AML. Schematic representation of mitochondrial metabolism highlighting OXPHOS as a central hub integrating glycolysis, TCA cycle, fatty acid oxidation, and purine synthesis in *FLT3*-ITD-mutated AML cells. Low spare respiratory capacity indicates mitochondrial vulnerability.

Mitochondria serve as cellular metabolic hubs within leukemic cells, where carbohydrate, amino acid, and fatty acid pathways converge in the tricarboxylic acid (TCA) cycle. (Figure 2) [49]. The key metabolic processes orchestrated by these organelles, including TCA cycle activity, glutaminolysis, oxidative phosphorylation, and fatty acid oxidation, are profoundly altered in AML [50].

Normal hematopoietic stem cells (HSCs) primarily rely on glycolysis for energy production, whereas leukemia stem cells (LSCs) depend heavily on oxidative phosphorylation (OXPHOS) for their biosynthetic processes and survival [51,52]. This distinction highlights OXPHOS as a potential novel target in AML. In particular, *FLT3*-mutated AML cells exhibit unique metabolic flexibility by rapidly activating OXPHOS upon oxygen availability, which is crucial for their survival in a fluctuating hypoxic and normoxic environment within the bone marrow [53,54,55]. The metabolic adaptability of leukemic cells, as they switch from anaerobic glycolysis to OXPHOS, enables efficient energy production and helps maintain mitochondrial outer membrane impermeability, which is essential for cell survival [56]. Such metabolic plasticity helps to develop resistance to chemotherapy. On the other end, these cells demonstrate a low spare respiratory capacity compared to their normal counterpart, suggesting that targeting the OXPHOS chain could be a promising therapeutic strategy for this specific patient subset [13,14,57]. Notably, OXPHOS and purine synthesis are central metabolic pathways targeted by different synthetic lethal treatments to resensitize leukemic cells to TKI treatment [58]. Indeed, recent studies in sorafenib-resistant *FLT3*-ITD cell lines have revealed that resistant leukemic cells undergo metabolic reprogramming characterized by impaired mitochondrial OXPHOS and a compensatory upregulation of glycolysis. This metabolic shift supports cell survival under sustained TKI pressure despite mitochondrial dysfunction [59,60]. Importantly, these resistant cells demonstrate collateral sensitivity to glycolytic inhibitors such as 2-deoxyglucose (2-DG) and 3-bromopyruvate propylester (3-BrOP), which target key glycolytic enzymes including hexokinase 2, highly expressed in the mitochondrial fraction of resistant cells [60,61,62].

Exploiting this metabolic vulnerability through synthetic lethal strategies, combining FLT3 tyrosine kinase inhibitors with glycolytic blockade, has been shown to resensitize resistant leukemic cells to TKI treatment, overcoming resistance mechanisms and promoting apoptotic cell death more effectively. [61,63,64] Furthermore, targeting mitochondrial oxidative phosphorylation (OXPHOS) and purine synthesis pathways alongside FLT3 inhibition has emerged as a promising complementary approach to enhance therapeutic efficacy [59]. Collectively, these findings underscore the critical role of metabolic rewiring in TKI resistance and provide a strong rationale for combined therapeutic strategies targeting both oncogenic signaling and metabolic dependencies to achieve durable responses in *FLT3*-mutated AML.

### 3.2. Aerobic Glycolysis

Beyond bioenergetics, glucose metabolism encompasses multiple pathways, including the pentose phosphate pathway, serine biosynthesis, and one-carbon metabolism, collectively generating essential molecular precursors [65]. AML cells demonstrate elevated GLUT1 expression, correlating with increased glucose uptake, while heightened pyruvate and lactate concentrations in patient sera are associated with unfavorable prognosis [66]. Over a decade ago, in mouse models, Ying-Hua Wang and colleagues established that genetic ablation of glycolytic enzymes PKM2 or LDHA disrupts aerobic glycolytic flux and attenuates leukemic expansion, whereas normal hematopoietic stem cells demonstrate tolerance to depletion of either enzyme. Paradoxically, normal cellular compartments showed increased proliferative capacity upon PKM2 or LDHA deletion [67]. Thus, the glycolytic pathway is important for leukemia maintenance and progression, and leukemic cells are more sensitive to the inhibition of AG than normal hematopoietic cells. AG remains fundamental in tumor proliferation, metastasis, and therapeutic resistance through multifaceted regulation of glycolytic enzymes, signaling cascades, non-coding RNAs, and bone marrow microenvironmental factors.

*FLT3*-mutated AMLs exhibit heightened glycolytic activity [68]. Despite increased lactate production, this does necessarily reduce mitochondrial pyruvate metabolism, which remains crucial for energy production in these cells [69,70]. *FLT3*-mutated AML cells display a unique metabolic profile characterized by low levels of pyruvate dehydrogenase kinase 1 (PDK1), which is involved in deactivating pyruvate dehydrogenase (PDH), a critical mitochondrial multienzyme complex responsible for catalyzing the oxidative decarboxylation of pyruvate, with this expression pattern correlating with heightened OXPHOS states. This metabolic profile is linked to a specific *FLT3*-mutated/PDP1 signaling axis that mediates pyruvate metabolism [71].

The increased glycolysis is regulated by key enzymes such as hexokinase 2 (HK2), which is involved in glucose phosphorylation and glycolytic pathway activation. *FLT3* mutations lead to constitutive activation of glycolysis through the activation of MYC, enhancing FLT3 expression and inducing a positive feedback loop that further amplifies glycolytic metabolism [68]. In this scenario, the interaction between the first glycolytic enzyme HK2 and the voltage-dependent anion channel 1 (VDAC1) on the outer mitochondrial membrane facilitates the coupling of glycolysis and OXPHOS, significantly increasing ATP production and supporting the high energy demands of leukemic cells [72]. Targeting this glycolytic pathway may impair the metabolic function of FLT3-ITD AML cells and provide potential therapeutic avenue.

Emerging research has uncovered distinctive metabolic characteristics in *FLT3*-mutated AML, particularly their enhanced aerobic glycolytic metabolism compared to wild-type counterparts [73]. Targeted metabolic interventions reveal a remarkable therapeutic vulnerability: glycolysis inhibition demonstrates selective cytotoxicity against *FLT3*-ITD leukemic cells while sparing wild-type leukemia and normal cellular populations [74]. Pre-clinical investigations in a murine model explored the synergistic potential of combined therapeutic strategies, specifically integrating a glycolysis inhibitor (2-deoxy-D-glucose) with the multi-targeted tyrosine kinase inhibitor sorafenib. The combination therapy demonstrated significantly prolonged survival compared to monotherapy, with experimental mice experiencing extended survival from 32 to 41 days.

### 3.3. Glutaminolysis

Standard amino acids support a wide range of cellular functions critical for neoplastic proliferation, including protein synthesis, nucleotide production, lipid biosynthesis, and glutathione generation. They also play key roles in the regulation of epigenetic mechanisms and post-translational modifications [75]. Glutamine represents the predominant amino acid supporting AML proliferation and survival through dual functionality in α-ketoglutarate provision for TCA cycling and leucine importation, facilitating mTORC1-mediated protein synthesis. Glutamine enters AML cells via SLC1A5 transporters before conversion to glutamate and α-ketoglutarate through glutaminolytic pathways, particularly clear in *FLT3*-mutated AML. Comparative analyses demonstrate heightened glutamine dependency in leukemic blasts versus normal HSCs, evidenced by resistance of normal HSCs to glutamine deprivation-induced apoptosis, contrasting with significant cell death in primary AML samples following SLC1A5 knockdown [76]. Furthermore, glutamine limitation substantially reduces oxygen consumption rates, suggesting critical interdependence between glutaminolysis and oxidative phosphorylation in leukemic metabolism [77].

Notably, glutamine metabolism emerges as another critical metabolic process in *FLT3*-mutated AML. Disrupting glutamine uptake, such as through the inhibition of SLC1A5, a transporter crucial for glutamine uptake, prevents mTORC1 activation and induces apoptosis, sparing normal HSCs. Experimental studies using shRNA-mediated inhibition of SLC1A5 showed antileukemic effects in xenograft mouse models, inducing autophagy and significantly reducing tumor growth [78]. Glutamine oxidation is particularly important for cells resistant to treatments like quizartinib, as these cells rely on mitochondrial metabolism for survival.

Mitochondrial glutamine catabolism represents a fundamental metabolic vulnerability in *FLT3*-ITD AML cells that exhibit therapeutic resilience to quizartinib [79]. These treatment-refractory malignant clones demonstrate pronounced reliance on glutaminolytic pathways to sustain bioenergetic homeostasis and biosynthetic processes, underscoring their dependency on oxidative phosphorylation machinery [79]. Compelling evidence reveals remarkable therapeutic synergy when quizartinib is paired with L-asparaginase, an amidohydrolase that depletes circulating glutamine and asparagine pools. This combinatorial strategy substantially attenuates glutamine catabolism in surviving leukemic populations, diminishing their capacity for metabolic persistence following FLT3 pathway blockade. Mechanistically, FLT3-ITD oncogenic signaling orchestrates upregulation of pyruvate dehydrogenase phosphatase 1 (PDP1), a key regulatory component of the pyruvate dehydrogenase multienzyme complex, through RAS-mediated transcriptional cascades. Elevated PDP1 expression augments both glycolytic flux and glutamine utilization, conferring metabolic flexibility that enables resistance to FLT3 inhibition. Notably, genetic ablation of *PDP1* restores cellular sensitivity to quizartinib even under oxygen-limited conditions, establishing PDP1 as a critical metabolic rheostat in *FLT3*-mutant leukemogenesis. The therapeutic rationale for quizartinib-L-asparaginase combination therapy extends beyond simple glutamine depletion, encompassing disruption of the enhanced oxidative metabolism that characterizes resistant cell populations. By simultaneously impairing mitochondrial amino acid oxidation and PDP1-driven metabolic reprogramming, this anti-metabolic intervention may eliminate minimal residual disease and augment treatment efficacy [80]. Indeed, the combination of quizartinib (AC220) with CB-839, a GLS inhibitor, leads to glutathione depletion, mitochondrial ROS accumulation, apoptotic cell death in vitro, and significantly extended survival in *FLT3*-ITD patient-derived xenograft models compared to FLT3 inhibition alone. CB-839 (telaglenastat), a highly selective and orally bioavailable GLS inhibitor, has shown promising tolerability in early-phase clinical trials across hematologic malignancies including AML, with mostly low-grade adverse events reported and an acceptable safety profile even in combination regimens [81]. By contrast, non-selective glutamine antagonists such as DON or acivicin historically induced dose-limiting gastrointestinal, neurological, and hematopoietic toxicities due to broad enzyme targeting [82,83]. Importantly, because immune effector cells (e.g., T cells, NK cells, macrophages) also rely on glutamine, GLS inhibition may carry the risk of immunosuppressive sequelae [82] (NCT02071927). Finally, the high metabolic plasticity of AML cells, encompassing upregulation of alternative substrate transporters or compensatory pathways like fatty acid oxidation or micropinocytosis, could limit long-term efficacy of glutaminase targeting, emphasizing the need for rationally designed combination regimens that balance metabolic targeting, antileukemic potency, and tolerability.

### 3.4. Fatty Acid Oxidation (FAO)

Lipids constitute an energetic substrate for neoplastic cells, not merely serving as energy reservoirs but providing essential components for membrane biogenesis and signaling pathway regulation [84,85]. AML blasts exhibit characteristic dysregulation of lipid metabolism compared with normal hematopoietic cells, presenting therapeutic targeting opportunities [86]. Comprehensive lipidomic profiling of plasma from AML patients at diagnosis revealed a striking pattern—there is a global reduction in total fatty acids and cholesterol, yet a selective elevation in certain free fatty acid species. These observations strongly imply a metabolic shift toward enhanced FAO as an adaptive response in AML metabolism [87]. In particular, the plasma of AML patients showed significantly increased levels of arachidonic acid (20:4 n − 6) and its precursors, such as gamma-linolenic acid (18:3 n − 6) and eicosatrienoic acid (20:3 n − 6), especially in individuals presenting with high bone marrow or peripheral blast counts and adverse prognostic risk [88]. These specific polyunsaturated fatty acids (PUFAs) are now recognized as metabolic biomarkers tightly associated with AML severity and metabolic reprogramming. Such selective enrichment of free fatty acids, despite overall plasma lipid depletion, underscores a proposed “futile cycle” in AML, whereby increased FAO provides acetyl-CoA to feed the TCA cycle and citrate pool, ultimately fueling de novo lipid synthesis required for rapid membrane biogenesis and proliferation. The upregulation of FAO-related transporters, such as carnitine transporter CT2 (SLC22A16), and enzymes like carnitine palmitoyltransferase I (CPT1a) further supports this metabolic rewiring as a pivotal survival strategy in AML cells [89]. This metabolic pathway supplies acetyl-CoA to the TCA cycle, subsequently increasing citrate production that initiates de novo fatty acid synthesis. FAO regulation plays a critical role in leukemic cell survival and quiescence, showing significant overexpression in AML cells compared to normal HSCs [90,91]. Quiescent HSCs maintain baseline FAO rates preserving dormancy, with metabolic status influencing symmetric versus asymmetric division outcomes and subsequent self-renewal capabilities [90,91].

It has been demonstrated that *FLT3*-mutated AML cells also showed increased fatty acid oxidation FAO rates, which play a pivotal role in supporting their metabolic needs [92]. Proteins such as CT2 and CPT1a are often upregulated, supporting this metabolic reliance. By promoting electron flux through the respiratory chain, agents like palmitate and dimethyl succinate can induce oxidative stress in *FLT3*-mutated cells, leading to cell death. This suggests that strategies promoting electron flux or targeting lipid metabolism, such as the PPARα agonist bezafibrate, known to increase mitochondrial mass and β-oxidation, may offer new therapeutic approaches for treating *FLT3*-mutated AML [93,94].

### 3.5. Sphingolipid Metabolism and Ceramide

Sphingolipids, including sphingomyelin (SM), ceramide (Cer), and glycosphingolipids, introduce another layer of metabolic complexity [95,96,97]. Sphingolipid formation and functionality depend upon oncogenic proteins, including sphingosine kinases and acid ceramidases, within AML cellular environments. Sphingosine-1-phosphate, generated through sphingosine kinase 1 activity, constitutively regulates AML cellular survival mechanisms [98]. Recent investigations demonstrate upregulation of S1PR3 (sphingosine-1-phosphate receptor 3) in both AML blasts and CD34+CD38- leukemic stem cells compared to normal hematopoietic stem cells. This receptor governs myeloid differentiation processes while activating inflammatory signaling cascades in primitive leukemic populations. Notably, S1PR3 activation in primary AML specimens promotes leukemic stem cell differentiation, potentially facilitating elimination of these therapy-resistant cellular reservoirs. Importantly, pharmacological activation of this receptor using the sphingosine-1-phosphate analog FTY720 (fingolimod) robustly reduced LSC frequency and leukemia burden in mice engrafted with patient-derived AML cells, including relapsed and chemoresistant samples. Strikingly, normal hematopoietic xenograft function was preserved, indicating a therapeutic window for selective targeting of LSCs. Therefore, harnessing S1PR3 signaling—either by genetic overexpression or via agonists such as FTY720—promises a strategy to deplete therapy-resistant leukemic reservoirs through enforced differentiation [99]. Moreover, FTY720 activates protein phosphatase 2A (PP2A) by disrupting SET–PP2A binding. In AML models, FTY720-mediated PP2A reactivation induces apoptosis and diminishes leukemic proliferation in a dose-dependent manner, with effects rescued by PP2A inhibition, indicating that the anticancer activity is PP2A-dependent [100,101]. Moreover, combining the CK2 inhibitor CX-4945 with FTY720 enhances antileukemic efficacy significantly. CX-4945 forces nuclear retention of SET, while FTY720 antagonizes SET–PP2A interaction in the cytoplasm, resulting in restored PP2A activity, reduced migration, and diminished cell viability and invasion in zebrafish AML xenografts—outperforming either agent alone [102].

Additionally, these lipid molecules not only just serve as structural components in cell membranes but also as potential regulators of cellular stress responses. Notably, Cer shows tumor-suppressing properties in many cancer types, and defects in Cer generation and clearance can lead to cancer cell survival and chemotherapy resistance [103,104]. The Cer transfer protein (CERT) transports Cer from the endoplasmic reticulum to the Golgi apparatus, playing a role in ceramide clearance and determining the ceramide-to-sphingomyelin ratio in cells. Inactivation of CERT has been shown to induce apoptosis in various cancer cell lines, including colon, breast, and lung carcinoma cells [105,106]. In *FLT3*-mutated AML, signaling pathways activated by *FLT3* mutations suppress ceramide synthase 1 (CerS1) and Cer metabolism [107]. Pharmacological inhibition of sphingosine kinase 1 (SPHK1) using agents like MP-A08 restores Cer levels, triggering integrated stress responses and sensitizing *FLT3*-ITD AML cells to apoptosis—particularly when combined with venetoclax [108,109]. Equally compelling is the targeted inhibition of the ceramide transfer protein CERT. Both genetic knockdown and pharmacological inhibition with HPA-12 selectively induces Cer retention in *FLT3*-ITD AML cells, reducing viability and promoting apoptosis while sparing *FLT3* wild-type cells [110]. Crucially, co-administration of HPA-12 with the FLT3 inhibitor crenolanib reveals a strong synergistic antileukemia effect, mediated through activation of the ER stress (GRP78/ATF6/CHOP axis) and induction of mitophagy [111,112]. This combination enhances Cer-driven apoptotic pathways and decreases leukemic stem cell reservoirs in both in vitro and in vivo models. Lipid accumulation within treated AML cells in different subcellular compartments suggests that lipids might induce other pro-cell death cascades, such as mitophagy [110]. Mitophagy has been reported to be involved in the inhibitory effect of crenolanib on *FLT3*-mutated AML cells. Another possibility is lipid stress-induced ferritin deficiency, particularly in *FLT3*-ITD AML. For instance, they can inhibit fatty acid uptake and oxidation, promoting intracellular lipid accumulation. In a recent study, fatty acid metabolism was inhibited through both genetic and pharmacological targeting of the FLT3–C/EBPα–SCD axis, including the use of APR-246 (eprenetapopt) to induce lipid peroxidation via glutathione depletion, and RSL3 to inhibit GPX4 and promote ferroptotic cell death. Although the specific SCD inhibitor was not disclosed, commonly used compounds such as A939572 or CAY10566 have been employed in similar contexts to suppress MUFA biosynthesis. Moreover, these interactions underscore the importance of ceramides as central nodes in lipid metabolism, affecting both the composition of cell membranes and cellular responses to variations in lipid levels [113]. These interactions underscore the importance of ceramides as central nodes in lipid metabolism, affecting both the composition of cell membranes and cellular responses to variations in lipid levels.

### 3.6. Anabolic Reprogramming in FLT3-ITD AML

Beyond conventional catabolic dependencies, *FLT3*-ITD-mutated AML demonstrates extensive metabolic reprogramming that encompasses amino acid utilization, one-carbon unit transfer reactions, and anabolic flux through the pentose phosphate pathway (PPP)—encompassing cellular processes fundamental to nucleotide biosynthesis, maintenance of redox homeostasis, and membrane component generation. Although specific investigations in *FLT3*-mutated disease remain sparse, comprehensive AML metabolomic analyses reveal enhanced expression of key enzymes including MTHFD2—a pivotal regulator of folate-mediated one-carbon transfer—alongside increased PPP activity, supporting cellular expansion under oncogenic conditions [114,115]. Additionally, l-type amino acid transporter 1 (LAT 1, SLC7A5) is frequently overexpressed, contributing to the uptake of branched-chain amino acids (BCAAs) and supporting oxidative respiration and anabolic growth. Notably, the selective LAT1 inhibitor JPH203 has demonstrated potent antileukemic activity in AML models, including samples resistant to venetoclax + azacitidine (Ven + Aza). Treatment with JPH203 impaired oxidative phosphorylation and viability in AML blasts, while sparing healthy hematopoietic cells; moreover, its combination with Ven + Aza produced synergistic eradication of leukemia activity in vitro [116].

The proliferative demands of AML cells necessitate sustained nucleotide and lipid production to fuel uncontrolled cellular division. These cells engage pentose phosphate pathway activation to produce ribose-5-phosphate precursors and generate NADPH, thereby enabling both de novo purine/pyrimidine synthesis and fatty acid production essential for membrane biogenesis. Recent evidence positions transketolase (TKT) as a central orchestrator of this metabolic rewiring: clinical specimens and established AML cell models exhibit substantially upregulated TKT levels, which facilitate cellular expansion, invasive capacity, and metabolic adaptation through transcriptional control of ribokinase (RBKS), creating a positive regulatory loop that amplifies non-oxidative pentose phosphate flux and promotes epithelial–mesenchymal transition phenotypes. In parallel, perturbation of guanine nucleotide biosynthesis, either through suppression of guanosine or pharmacological inhibition of Inosine Monophosphate Dehydrogenase 2 (IMPDH2), has been shown to induce myeloid differentiation and impair the oncogenic Lens Epithelium Derived Growth Factor (LEDGF)/menin/MLL-fusion complex in AML and MLL-rearranged leukemias. Notably, clinical translation of these insights includes the RNA polymerase I inhibitor CX-5461, which selectively downregulates menin and LEDGF, induces AML cell differentiation, and, critically, has demonstrated acceptable tolerability without neutropenia in early-phase hematologic trials [117].

Additionally, TKTL1, a transketolase paralog, mediates hypoxic adaptation in THP-1 leukemic cells through modulation of Glucose-6-Phosphate Dehydrogenase (G6PD) and Glyceraldehyde-3-Phosphate Dehydrogenase (GAPDH) enzymatic activity, maintaining both NADPH availability and glycolytic flux during environmental stress conditions [118]. Such anabolic dependencies present exploitable therapeutic windows: ribosomal assembly inhibitors and antifolate compounds that disrupt nucleotide availability have demonstrated promising activity in AML experimental systems and early clinical investigations.

Moreover, *FLT3*-ITD AML displays remarkable metabolic flexibility, deploying alternative nutrient acquisition mechanisms including bulk endocytosis (macropinocytosis), enhanced amino acid transporter expression, and metabolic shifts toward fatty acid oxidation when primary carbon sources become limiting [119]. This adaptive capacity undermines the sustained efficacy of single-pathway metabolic inhibitors. Notably, fatty acid oxidation-driven metabolic reprogramming contributes to resistance against venetoclax or FLT3-targeted agents, emphasizing the requirement for coordinated targeting of both catabolic and biosynthetic networks to prevent metabolic escape.

Collectively, therapeutic metabolic intervention in *FLT3*-ITD AML must extend beyond targeting glutamine catabolism or oxidative phosphorylation to encompass disruption of biosynthetic networks—encompassing nucleotide, membrane lipid, and amino acid production—through strategically designed combination approaches. This framework requires biomarker-guided patient selection, precision inhibitors targeting specific enzymes (such as MTHFD2, pentose phosphate regulators, or amino acid carriers), and optimized pharmacological delivery systems to achieve maximal antileukemic efficacy while preserving metabolic function in healthy proliferating tissues including immune effector cells.

### 3.7. ROS Dynamics in FLT3-Mutated AML

As discussed above, mitochondrial dependency recently emerged as a pivotal driver of energy in AML pathogenesis and progression. Notably, LSCs showed an increased mitochondrial mass and O_2_ consumption rate as compared to normal HSCs [120]. Indeed, OXPHOS dependency is closely linked to the increased production of mitochondrial reactive oxygen species (mtROS), which play a wide range of roles, including stimulating signaling pathways that promote tumorigenesis. Specifically, JNK, activated by ROS and endoplasmic reticulum stress (e.g., via IRE1α), is required for survival of *FLT3*-ITD leukemia cells, and its pharmacologic inhibition using SP600125 induces apoptosis even in TKI-resistant *FLT3*-ITD-TKD blasts [121]. While ERK is engaged simultaneously, ensuring complementary regulation of survival and proliferation pathways in *FLT3*-ITD AML, driven by RAS/MEK signaling downstream of *FLT3*-ITD, it promotes leukemic proliferation and metabolic remodeling and contributes to adaptive resistance following FLT3 inhibitor therapy [122]. Collectively, JNK/ERK, HIF1α, and associated regulatory networks orchestrate mitochondrial biogenesis and the regulation of protein function through various post-translational modifications [123]. During cellular metabolism, electrons derived from NADH and FADH_2_, which are products of substrate oxidation including glucose, glutamine, and fatty acids, are transferred through the ETC complexes, establishing a proton gradient essential for ATP synthesis [124,125]. During the process, however, either proton or electron leakage occurs. Proton and electron leak balance is intricately linked with superoxide production and cellular metabolism and resistance to stress. Electron leakage, predominantly occurring at Complex I (NADH dehydrogenase) [126] and Complex III (cytochrome bc_1_ complex), results in the partial reduction of molecular oxygen to form superoxide (O_2_^−^) [127]. The generated superoxide is subsequently converted to hydrogen peroxide (H_2_O_2_) through the action of mitochondrial superoxide dismutase SOD1 in the intermembrane space and SOD2 in the matrix [128]. H_2_O_2_ can traverse membranes, functioning either as a signaling molecule or inducing oxidative damage to cellular components.

Previous investigations have established that *FLT3* mutations contribute to oxidative damage through multiple mechanisms. One significant pathway involves increased intracellular reactive oxygen species (ROS) production in *FLT3*-mutated cells compared to their wild-type *FLT3* counterparts, with NADPH oxidases (NOX family) localized in the endoplasmic reticulum and mitochondria serving as primary ROS sources [129]. While physiological ROS levels play crucial roles in cellular signaling and homeostasis, elevated concentrations rapidly react with biomolecules including proteins, lipids, carbohydrates, and nucleic acids, resulting in irreversible functional alterations or complete molecular destruction [130]. Additionally, *FLT3* mutations hyperactivate the STAT5 and PI3K/AKT signaling cascades, thereby maintaining or enhancing the expression of p22phox and NOX proteins [131,132]. This upregulation promotes ROS production that subsequently diffuses into the nucleus, causing DNA damage—particularly double-strand breaks and mismatches. Additionally, the localization of FLT3 at the plasma membrane is essential for maintaining NOX protein levels and preventing the GSK3-β-mediated proteasomal degradation of p22phox. The functional mechanism involves NOX proteins associating with p22phox for membrane co-stabilization, followed by RAC1 binding and GDP-GTP exchange, which initiates oxygen conversion to superoxide [15].

Notably, *FLT3*-mutated cells exhibit increased RAC1-GTP binding to phosphorylated STAT5, resulting in enhanced recruitment of RAC1-GTP to NADPH oxidase complexes. Of particular significance is the nuclear membrane-bound NOX4D isoform, which is highly expressed in *FLT3*-ITD-positive patients and cell lines but nearly absent in wild-type *FLT3*-expressing counterparts. This isoform generates ROS that promote leukemic cell survival. A feedback loop exists between FLT3 signaling and ROS production, creating a self-reinforcing cycle. The oxidative environment enhances both wild-type FLT3 and FLT3-ITD signaling, possibly through oxidation of specific cysteine residues such as Cys790, which, in turn, further increases ROS production [133].

Recent research by Wu and colleagues demonstrated that in *FLT3*-mutated AML, FLT3 signaling upregulates key DNA damage response (DDR) factors through distinct pathways [134]. Through STAT5 activation, FLT3-ITD enhances expression of Wee1-like protein kinase (WEE1), checkpoint kinase 1 (CHK1), and proviral integration site for Moloney murine leukemia virus-1 (PIM-1). Concurrently, via ERK pathway activation, FLT3-ITD increases expression of radiation sensitive 51 (RAD51) and mismatch repair (MMR) factors including MutS homolog 2 (MSH2), MSH6, and MutL homolog 1 (MLH1). These adaptive responses promote AML cell survival under oxidative stress conditions and contribute to chemoresistance. Metabolically, *FLT3*-ITD-expressing cells exhibit distinctive characteristics, including elevated expression of succinate-CoA ligases and enhanced mitochondrial electron transport chain (ETC) complex II activity. This heightened respiratory capacity correlates with increased mitochondrial metabolism and consequent ROS production. Indeed, *FLT3* mutations drive specific gene expression signatures [135], and *FLT3*-mutated AMLs typically display increased ROS levels, leading to enhanced DNA double-strand breaks [136]. Moreover, inhibition of FLT3-ITD creates a specific dependency on glutaminolysis for cellular survival [137].

Recent comprehensive multi-omics analyses by Erdem et al. have stratified AML based on pyruvate dehydrogenase kinase 1 (PDK1) expression levels [71]. PDK1 was identified as a critical determinant of distinct metabolic states in AML. PDK1-high AMLs exhibit reduced mitochondrial oxidative phosphorylation and frequently retain wild-type *FLT3* and *NPM1*, also displaying enriched stemness signatures. Conversely, PDK1-low AMLs commonly harbor *FLT3* mutations and are characterized by elevated cell cycle activity, enhanced oxidative phosphorylation, L-GMP (lymphoid-primed multipotent progenitor) signatures, and the highest oxygen consumption rates.

Kannan et al. proposed targeting the NRF2/HO-1 antioxidant pathway in *FLT3*-mutated AML to enhance therapeutic efficacy [138]. In non-*FLT3* mutant AML, heme oxygenase-1 (HO-1) contributes to resistance against tumor necrosis factor (TNF)-induced apoptosis and epigenetically targeted agents [139]. The transcription factor NRF2, a major driver of HO-1 expression, has also been implicated in drug resistance in AML [140]. While numerous clinical studies have targeted NRF2 across various cancer types over the past three decades, none have specifically focused on *FLT3*-mutant AML [141,142], despite evidence suggesting that targeting NRF2 and antioxidant pathways may deplete leukemic stem cells (LSCs) [143,144]. Furthermore, there remains a need for reliable biomarkers of NRF2 inhibition across cancer types, with recent data suggesting that HO-1 expression or downstream redox parameters such as ROS levels or glutathione (GSH/GSSG) ratios may serve as useful indicators in *FLT3*-mutated AML.

### 3.8. Metabolic Targeting: Balancing Antileukemic Efficacy and Normal Cell Function

A critical challenge in metabolic targeting is posed by cancer stem cells (CSCs), a rare and intrinsically resistant subpopulation first identified in AML and later in various malignancies that sustains long-term tumor propagation through self-renewal and multilineage differentiation. While driving both drug resistance and therapy tolerance. These dual properties are driven by mechanisms such as quiescence-associated drug insensitivity, activation of survival pathways, and evasion of apoptosis [61,63,64,145,146]. Importantly, CSCs exhibit cell cycle heterogeneity and may persist as either resistant or tolerant clones under therapeutic pressure, complicating efforts to eradicate the disease through conventional or metabolic interventions.

This challenge is further deepened by the fact that canonical metabolic reprogramming events including enhanced glycolytic flux, augmented oxidative phosphorylation capacity, and glutamine catabolism are not restricted to malignant cells but represent conserved features of non-malignant proliferative cellular states, including activated T lymphocytes, tissue-resident macrophages, and other immune effector populations. Upon antigenic stimulation, T cells rapidly upregulate GLUT1-mediated glucose transport and ASCT2-dependent glutamine uptake, engage aerobic glycolysis concurrent with mitochondrial respiration, and activate mTORC1 signaling to satisfy biosynthetic and bioenergetic requirements—metabolic signatures that mirror those observed in transformed cells (including Warburg-type glycolysis and glutamine-fueled anaplerosis). Consequently, pharmacological disruption of glutamine metabolism or glycolytic pathways may compromise the activation kinetics, proliferative capacity, or effector functions of essential immune cell subsets, including CD8+ T cells, NK cells, B lymphocytes, and M1 macrophages, which exhibit obligate glutamine dependence for cytokine biosynthesis and antimicrobial responses [147,148,149].

From a translational perspective, broad-spectrum glutamine analogs such as 6-diazo-5-oxo-L-norleucine (DON) or L-(αS,5S)-α-amino-3-chloro-4,5-dihydro-5-isoxazoleacetic acid (acivicin)—which inhibit multiple glutamine-utilizing enzymes—have demonstrated dose-limiting toxicities including severe enterocolitis, peripheral neuropathy, and myelosuppression, reflecting their indiscriminate targeting of glutamine-dependent processes in rapidly dividing normal tissues [150,151]. While selective allosteric glutaminase (GLS1) inhibitors such as CB-839 (telaglenastat) and bis-2-(5-phenylacetamido-1,3,4-thiadiazol-2-yl)ethyl sulfide (BPTES) exhibit enhanced tumor selectivity and improved therapeutic windows, the metabolic glutamine addiction of immune effector cells poses inherent risks of treatment-related immunosuppression, particularly under conditions of prolonged GLS1 blockade [152,153]. Furthermore, the metabolic adaptability characteristic of AML blasts, including compensatory activation of alternative nutrient scavenging mechanisms (macropinocytosis, upregulation of amino acid transporters such as LAT1/SLC7A5) or metabolic rewiring toward fatty acid β-oxidation, presents significant challenges for durable therapeutic responses to single-agent metabolic inhibitors [154]. These considerations underscore the imperative for rational combination strategies or tumor-selective delivery platforms designed to maximize on-target antileukemic activity while preserving physiological metabolic functions in normal proliferative compartments.

In summary, effective metabolic intervention in *FLT3*-ITD AML necessitates comprehensive evaluation of potential off-target effects in normal proliferative tissues, particularly immune surveillance mechanisms, and implementation of precision medicine approaches incorporating biomarker-driven patient stratification, target-selective inhibition, pharmacokinetically optimized dosing regimens, and synergistic combination therapies that achieve maximal therapeutic efficacy while maintaining acceptable toxicity profiles.

Despite the challenges of balancing antileukemic efficacy with preservation of normal cellular metabolism, the identification of specific metabolic vulnerabilities of *FLT3*-mutated leukemia cells opens new avenues for targeted therapeutic strategies. In the following section, we will explore these distinct metabolic dependencies as promising targets for more selective and effective treatment.

## 4. Metabolic Vulnerabilities of *FLT3*-Mutated AML

With growing evidence on the dynamic nature of leukemic cell metabolism, the identification of specific metabolic dependencies paved the way for the design of metabolic-oriented treatments able of disarming leukemic cells and potentially overcoming challenges associated with drug resistance.

For many years, intensive induction chemotherapy has represented the standard of care for de novo AML, despite carrying high rates of early post-induction deaths and limited therapeutic benefit for elderly patients [155,156]. Accordingly, the exploration of less toxic treatment options, such as hypomethylating agents (HMAs), has significantly improved overall survival (OS) in patients not eligible for intensive induction therapy. Among the emerging trends in AML treatment, several targeted agents, including inhibitors of FLT3 (FLT3i) [36], Bcl-2 [35], and IDH1/2 [157,158], as well as gemtuzumab ozogamicin [159] and CPX351 [160], have radically changed treatment options for AML. The therapeutic landscape of *FLT3*-mutated AML has been significantly improved with the approval of tyrosine kinase inhibitors (TKIs), which, either as a single agent or in combination with other drugs, offer less aggressive options and, in some cases, avoid the need for cytotoxic chemotherapy [35]. However, despite these developments, many patients still develop resistance, highlighting the urgent need for innovative approaches.

In the last decade, increasing knowledge about the contribution of metabolic rewiring in AML pathogenesis and progression has led to the investigation of various metabolic interventions, with the aim to further improve treatment outcomes and address resistance mechanisms. Importantly, metabolic rewiring has been extensively observed across various AML subtypes, independent of the presence of *FLT3* alterations [161]. Indeed, each AML subtype is associated with a distinct molecular profile and phenotypic landscape that shapes its metabolic dependencies. For instance, metabolomic analyses revealed that *NPM1*-mutated AML is characterized by high levels of free carnitine, whereas *FLT3*-ITD displays significant alterations in amino acid metabolism, particularly involving glutamic and aspartic acids, highlighting the key role of glutaminolysis and energy metabolism pathways in differentiating these subtypes [162]. Additionally, IDH1/2 mutations enhance mitochondrial respiration and fatty acid β-oxidation [163], while TP53 [164], DNMT3A [165], and ASXL1 mutations [166] drive distinct metabolic alterations, including enhanced glycolysis, TCA cycle remodeling, mitochondrial overactivation via the Akt/mTOR pathway, and increased oxidative stress, ultimately contributing to hematopoietic stem cell dysfunction and leukemogenesis.

These shared and subtype-specific metabolic vulnerabilities suggest that many metabolic-targeted therapies hold potential beyond *FLT3*-mutated AML. However, patient stratification integrating metabolic and genomic profiling could represent an excellent strategy to maximize therapeutic efficacy and to design approaches aimed at overcoming resistance mechanisms.

A number of metabolic-oriented drugs have been developed and tested in AML, either pre-clinically or in early-phase trials. These agents target key metabolic processes, offering potential to overcome treatment resistance and selectively eradicate LSCs (Table 1). Notably, treatment resistance in *FLT3*-mutated AML is closely related to dynamic metabolic adaptations that evolve over time. LSCs harbor metabolic plasticity, enabling them to shift between energy pathways to survive therapeutic pressure. Early in treatment, *FLT3*-mutated AML cells rely heavily on mitochondrial metabolism and OXPHOS. However, under prolonged FLT3i exposure, these cells adapt by increasing glycolysis and glutaminolysis to maintain energy production and redox balance, contributing to drug resistance [167,168,169].

A recent study by van Gils et al. categorizes metabolic dysregulation as one of six key mechanisms involved in therapy resistance in LSCs [180]. Notably, LSCs often reside in a quiescent state characterized by low ROS levels, favoring low-energy metabolic pathways that limit the efficacy of conventional chemotherapies, which target rapidly proliferating blasts. Additionally, metabolic heterogeneity exists among AML subsets, with some proliferative blasts maintaining mitochondrial activity but exhibiting reduced sensitivity to treatment, highlighting metabolic adaptation as a crucial factor in resistance [181]. These metabolic dynamics and their evolution over time emphasize the need for therapeutic strategies that target multiple metabolic pathways in *FLT3*-mutated AML to overcome resistance and improve patient outcomes.

Interestingly, bone marrow stromal cells showed the ability to transfer functional mitochondrial to AML cells through contact-dependent endocytic pathways, enhancing mitochondrial ATP production and survival under chemotherapy stress. This mitochondria-mediated metabolic support is particularly relevant for leukemia-initiating cells, contributing to treatment resistance and disease relapse [182].

Recent studies have clearly demonstrated that *FLT3*-ITD-mutated leukemic cells heavily rely on mitochondrial metabolism to maintain redox homeostasis and that FLT3 inhibition disrupts this balance by reducing OXPHOS activity, leading to decreased ATP production and an increased reliance on glycolysis, making these cells highly sensitive to oxidative stress and mitochondrial damage [183]. Additionally, FLT3i disrupt de novo purine synthesis, which is strictly dependent on mitochondrial function [58].

Several studies have highlighted the potential of combination treatments that target and disrupt metabolic pathways in AML treatment, as summarized in Table 2. These strategies focus on exploiting metabolic vulnerabilities in AML cells. One notable example of synthetic lethality involved the combination of the FLT3i Quizartinib (AC220) with glutaminase inhibitors, such as CB-839, and IACS-010759, a small-molecule inhibitor of complex I of the electron transport chain (ETC), offering a more effective and less toxic therapeutic approach [184,185]. Additionally, recent studies have shown that combining FLT3i with PDP1 inhibition reduces OXPHOS activity and enhances AML sensitivity to FLT3i, further supporting the promise of combination therapies targeting metabolic vulnerabilities in *FLT3*-mutated AML. Indeed, FLT3-ITD signaling upregulates PDP1 via RAS-MAPK, activating the pyruvate dehydrogenase complex and directing pyruvate toward mitochondrial oxidation. PDP1 depletion selectively impairs respiration and proliferation in *FLT3*-ITD cells while sparing wild-type counterparts. Crucially, PDP1 mediates quizartinib resistance by sustaining OXPHOS following FLT3 inhibition, and its knockdown restores drug sensitivity [80]. Moreover, FLT3 inhibition primarily disrupts glucose utilization and glycolytic pathways, while sparing glutamine metabolism, thereby inducing a significant dependence on glutaminolysis to sustain mitochondrial function and TCA cycle [186]. Accordingly, targeting glutaminolysis through GLS inhibitors emerges as a potential therapeutic strategy to overcome resistance to FLT3i. In line with these pre-clinical findings, several ongoing and completed clinical trials are investigating the efficacy of combining FLT3i with metabolic agents in relapsed/refractory AML. In particular, the FRIDA trial (NCT05546580) is evaluating the combination of Gilteritinib with ladademstat, an LSD1 inhibitor known to affect mitochondrial metabolism and epigenetic regulation. Additional studies are exploring regimens that include Venetoclax (NCT03625505, NCT04140487); Selinexor (NTC02530476), a nuclear export inhibitor that disrupts energy production and glycolysis; or hypomethylating agents, which interfere with mitochondrial metabolism and redox homeostasis.

Among metabolic-oriented drugs, Venetoclax in combination with azacitidine represents the standard of care for patients with newly diagnosed AML who are unfit for intensive chemotherapy.

Several studies have widely investigated the strong metabolic reprogramming induced by Venetoclax, through the inhibition of mitochondrial metabolism with an effect independent of Bcl-2 inhibition. Furthermore, combination with azacitidine disrupts energy metabolism by reducing glutathione levels, impairing OXPHOS and ultimately targeting leukemia stem cells (LSCs) [187]. The synergistic effect of Venetoclax and azacitidine further highlights the potential of mitochondrial metabolism inhibition in overcoming metabolic vulnerabilities in AML. Notably, a synergistic effect with FLT3i has been demonstrated through the suppression of MCL-1, a pro-survival protein with a key role in leukemia cell survival, especially in high-risk AML cases [195,196].

In light of the results obtained from targeting mitochondrial metabolism, further investigations have been conducted to test the in vitro and in vivo effects of mitochondrial ETC inhibitors, which have been revealed to be effective at reducing leukemia cell viability. Indeed, the high activity of Complex II led to the testing of the effect of genetic knockdown of the chaperone SDHAF1, which was able to delay the growth of AML cells. Consistent with this, pharmacological inhibition of Complex III using antimycin A resulted in a marked decrease in cell proliferation and triggered differentiation processes [188]. Lastly, the inhibition of mitochondrial ATP-synthase (complex V) with oligomycin A significantly improved the sensitivity of *FLT3*-mutated AML to FLT3i [189]. Overall, these findings may contribute to the identification of novel mitochondrial metabolic targets that could be exploited in combination with established therapies, such as venetoclax, to enhance treatment efficacy and potentially reduce toxicity.

A widely used drug with pleiotropic effects on metabolism is metformin, which has also been tested as an adjuvant treatment for leukemia. Metformin is able to inhibit OXPHOS in AML cells, also impairing glycolysis, and it induces apoptosis in AML cells without affecting normal hematopoietic stem cells [190]. Additionally, it enhances the effectiveness of FLT3i like sorafenib, potentially overcoming resistance by targeting the mTOR pathway, and the sensitivity to venetoclax, with a synergistic effect which results in greater antileukemia effects [191,197]. Moreover, *FLT3*-positive cells with acquired quizartinib resistance have been the subject of metabolic adaptation studies. Researchers in France discovered that these resistant leukemic cells become dependent on mitochondrial metabolism, with a specific reliance on glutamine oxidation pathways for their continued survival. Their investigation revealed a promising synergistic relationship between quizartinib and L-asparaginase that functions through complementary anti-metabolic mechanisms [79].

Additionally, Sphingolipids, including sphingomyelin (SM), ceramide (Cer), and glycosphingolipids, introduce another of metabolic target. In *FLT3*-mutated AML, signaling pathways activated by *FLT3* mutation suppress ceramide synthase 1 (CerS1) and ceramide metabolism. Targeting *FLT3*-mutated AML with the FLT3 inhibitor crenolanib induced ceramide accumulation, leading to AML cell death. Additionally, targeting sphingosine kinase 1 (SPHK1) with its inhibitor MP-A08 induced ceramide accumulation, activated the downstream apoptotic integrated stress response, and sensitized AML cells to venetoclax.

Indeed, lipid accumulation within treated AML cells in different subcellular compartments suggests that lipids might induce other pro-cell death cascades, such as mitophagy. Mitophagy has been reported to be involved in the inhibitory effect of Crenolanib, an FLT3 inhibitor, inducing Cer accumulation and subsequently promoting cell death in *FLT3*-mutated AML cells [198,199].

The high dependency of *FLT3*-mutated cells on glycolysis has resulted in the identification of key glycolytic enzymes, including hexokinase 2 (HK2) and pyruvate kinase M2 (PKM2), which are often upregulated in this AML setting. Indeed, the use of glycolytic inhibitors, such as 2-Deoxy-d-Glucose (2-DG), significantly enhances the cytotoxic effects induced by TKIs [192,193]. Pharmacological inhibition of the de novo serine synthesis pathway with phosphoglycerate dehydrogenase (PHGDH) inhibitors, such as WQ-2101, significantly enhances the sensitivity of *FLT3*-mutated AMLs to standard chemotherapy [194]. In particular, PHGDH demonstrates marked overexpression in AML populations characterized by therapeutic refractoriness and adverse clinical outcomes. Genetic ablation or small-molecule inhibition of PHGDH selectively attenuates proliferative capacity and triggers programmed cell death in *FLT3*-ITD leukemic models both in vitro and within xenograft systems. Emerging evidence reveals that *FLT3*-ITD-positive patients exhibit systematic upregulation of serine metabolic machinery, encompassing downstream enzymes PSAT1 and PSPH, establishing a metabolic signature associated with enhanced serine flux. Notably, PHGDH inhibition demonstrates synergistic antileukemic activity when combined with established therapeutic agents, including Rylaze and the nucleoside analog cytarabine, suggesting that serine biosynthetic dependency represents an exploitable metabolic vulnerability in *FLT3*-mutant AML.

Overall, the growing understanding of metabolic rewiring and the identification of distinct metabolic vulnerabilities in *FLT3*-mutated AML have opened new avenues for novel therapeutic approaches, supporting the rationale for combining FLT3 inhibitors with agents targeting mitochondrial metabolism, glycolysis, glutaminolysis, and lipid pathways. While these insights underscore the potential of metabolic biomarkers in designing personalized treatment strategies, their clinical application still faces significant challenges. Metabolic plasticity, clonal heterogeneity, and the limited clinical validation of these biomarkers remain critical barriers to their effective translation into durable, patient-tailored therapies. Further exploration integrating genomic, metabolomic, and functional profiling will be essential to refine patient stratification and advance the development of metabolism-oriented therapeutic paradigms in AML.

## 5. Conclusions

The advances in understanding *FLT3*-positive clone metabolism underscore the importance of a translational approach that integrates metabolomics, genomics, and pharmacology to develop personalized therapies. Mitochondrial metabolism and its associated pathways are central therapeutic vulnerabilities in *FLT3*-mutated AML. The identification of metabolic dependencies has spurred the development of combinatorial approaches targeting OXPHOS, glycolysis, glutamine, and lipid metabolism, as well as ROS-regulated signaling. Notably, redox-sensitive markers such as HO-1 and PDK1 are emerging as potential biomarkers for patient stratification and treatment response. The integration of these metabolic insights into clinical strategies promises to enhance therapeutic efficacy, overcome drug resistance, and, ultimately, improve patient outcomes. Continued research into the metabolic landscape of *FLT3*-mutated AML will be essential to refine targeted therapies and to develop personalized, metabolism-oriented treatment paradigms.

## Figures and Tables

**Table 1:** Emerging therapeutic approaches targeting metabolism in AML.

Drug	Metabolic Target	Trial	References
2-Deoxy-D-glucose (2-DG)	Glycolysis	Pre-clinical (in vitro and in vivo)	[68]
Atovaquone	OXPHOS	Pre-clinical (in vitro and in vivo) Feasibility Trial (NCT03568994)	[170]
Avocatin B, Etomoxir and ST-1326	Fatty acid oxidation	Pre-clinical (in vitro and in vivo)	[57,92,171]
3-bromopyruvate (3BrPA)	Glycolysis	Pre-clinical (in vitro and in vivo)	[66]
LCL204	Sphingolipids	Pre-clinical (in vivo)	[172]
IACS-010759	OXPHOS	Phase I (NCT02882321)	[173]
Telaglenastat (CB-839)	Glutaminolysis	Phase I (NCT02071927)	[174]
Tigecycline	OXPHOS	Phase I (NCT01332786)	[175]
BCT-100	Arginine metabolism	Phase I/II (NCT03455140)	[175]
ADI-PEG 20	Arginine metabolism	Phase II trial (NCT01910012)	[176]
Venetoclax	OXPHOS	Phase I/II/III (NCT01994837, NCT02203773, NCT02993523, NCT04801779, NCT05177731, NCT05048615, NCT03586609, NCT03625505, NCT04140487 NCT03455504)	[177,178]
Iadademstat	OXPHOS	NCT05546580	[179]

**Table 2:** Metabolic-oriented therapeutic strategies in *FLT3*-ITD-mutated AML.

Drug/Combination	Targeted Metabolic Pathway	Mechanism of Action	Effect	References
Quizartinib (1–5 nM) + CB-839	Glutaminolysis	Inhibits glutamine metabolism; increased ROS and mitochondrial dysfunction	Synergistic lethality; overcomes resistance mechanisms (in vitro and in vivo)	[79]
Quizartinib (100 nM) + IACS-010759 (10 nM)	OXPHOS (Complex I inhibition)	Inhibits mitochondrial electron transport chain Complex I	Enhances sensitivity to FLT3i; impairs energy production (in vitro)	[184]
Venetoclax (100 nM in vitro/100 mg/kg in vivo/400 mg/die in clinical trial) + Azacitidine (1 µM in vitro/3 mg/kg in vivo/75 mg/7 days in clinical trial)	Mitochondrial metabolism, Glutathione synthesis	Impairs OXPHOS, reduces GSH levels, inhibits Bcl-2	Targets LSCs; induces apoptosis; synergizes with FLT3i via MCL-1 suppression (in vitro, in vivo, and clinical trial)	[187]
Antimycin A (1 µM)	OXPHOS (Complex III inhibition)	Blocks electron flow in ETC Complex III	Reduces cell viability; promotes differentiation (in vitro)	[188]
Oligomycin A (0.5–10 nM in vitro/100 µg/kg in vivo)	OXPHOS (Complex V/ATP synthase inhibition)	Inhibits ATP production	Enhances sensitivity to FLT3 inhibitors (in vitro and in vivo)	[189]
Metformin (0.5–10 mM in vitro/200 mg/kg in vivo)	Mitochondrial metabolism/mTOR pathway	Inhibits OXPHOS and glycolysis; activates AMPK	Promotes apoptosis; synergizes with FLT3i and venetoclax (in vitro and in vivo)	[190,191]
Quizartinib + L-Asparaginase (2 UI/mL)	Amino acid metabolism (glutamine/asparagine depletion)	Depletes glutamine/asparagine; metabolic stress	Effective in quizartinib-resistant *FLT3*+ cells (in vitro)	[79]
Crenolanib (6 µM in vitro/15 mg/Kg/die in vivo) + MP-A08 (SPHK1 inhibitor, 6–20 µM/100 mg/kg)	Sphingolipid metabolism (ceramide pathway)	Induces ceramide accumulation; activates stress/apoptotic signaling	Promotes mitophagy and apoptosis; sensitizes to venetoclax	[110]
2-Deoxy-D-glucose (2-DG, 20 mM)	Glycolysis	Inhibits hexokinase; blocks glucose utilization	Enhances cytotoxic effects of TKIs (in vitro and in vivo)	[192,193]
WQ-2101 (PHGDH inhibitor)	Serine synthesis	Inhibits de novo serine synthesis pathway	Enhances chemotherapy response in *FLT3*-mut AML (in vitro and in vivo)	[194]

## Data Availability

No new data were created or analyzed in this study. Data sharing is not applicable to this article.
